# Structural and functional insights into *Pseudomonas aeruginosa’s* secretion systems 1–6: regulation, role in microbial keratitis and drug targets

**DOI:** 10.1007/s10096-025-05295-2

**Published:** 2025-10-18

**Authors:** Tanzina Akter, Paula-Maree Challita, Abrar Maswood Haider, Shiful Islam, Kaniz Fatema, Fiona Stapleton, Mark Willcox

**Affiliations:** 1 School of Optometry and Vision Science, Faculty of Medicine and Health, University of New South Wales (UNSW), Sydney, NSW 2052 Australia; 2 Microbial Biotechnology Division, National Institute of Biotechnology (NIB), Dhaka, 1349 Bangladesh; 3 Department of Microbiology, Primeasia University, Banani, Dhaka, 1213 Bangladesh; 4 Department of Biotechnology, Faculty of Natural Science, Norwegian University of Science and Technology, Trondheim, 7034 Norway

**Keywords:** *Pseudomonas aeruginosa*, Secretions systems, Virulence factors, Microbial keratitis, Anti-virulence strategies

## Abstract

**Background:**

*Pseudomonas aeruginosa* is a versatile Gram-negative pathogen that thrives in diverse environments. This pathogen causes a range of infections, including microbial keratitis (MK), a sight-threatening corneal infection. Central to its virulence are six specialized protein secretion systems (Types 1-6) that span the complex cell envelope. One-step systems (Types 1, 3, 4, 6) directly translocate effectors across both of its cell membranes, while two-step systems (Types 2, 5) first export substrates into the periplasm via the Sec or Tat pathways before outer-membrane release. These molecular machines deliver toxins, enzymes and competitive effectors that facilitate tissue damage, immune evasion, nutrient acquisition, biofilm formation and interbacterial killing. Their expression and activity are often coordinated by quorum-sensing networks (Las, Rhl, Pqs, Iqs). Although various secretion systems and their effectors have been characterized, the specific contributions of each system to corneal infection have yet to be comprehensively reviewed.

**Purposes and Methods:**

PubMed and Google Scholar were searched to synthesize current knowledge of the structure, regulation, and substrates of these secretion systems, to highlight their contributions to keratitis pathogenesis, and to evaluate emerging anti-virulence strategies targeting these pathways as novel therapeutics.

**Results:**

The T3SS system and its effectors ExoU and ExoS play dominant roles in keratitis severity while other secretion systems further enhance virulence by facilitating toxin release, biofilm development, and interbacterial competition. Regulatory interactions with quorum-sensing pathways amplify their impact during infection.

**Conclusion:**

Understanding the functional roles of all six secretion pathways and their regulatory mechanisms will be critical for identifying and developing novel anti-virulence therapeutics for MK.

## Introduction

*Pseudomonas aeruginosa* is a Gram-negative, rod-shaped, motile opportunistic human pathogen. It is commonly found in natural habitats such as soil, water, as well as hospital environments. One of the remarkable abilities of *P. aeruginosa* is that it can colonize in both abiotic surfaces and host tissues [1]. *P. aeruginosa* is a common cause of cystic fibrosis and other chronic lung infections, ventilator-associated pneumonia (VAP), burns and wound infections, urinary tract infections, bacteremia, and microbial keratitis (a serious ocular infection that can lead to vision loss if untreated) especially in immunocompromised patients [2–5]. Strains can often be multidrug-resistant (MRD) and armed with a sophisticated arsenal of molecular weapons posing a significant threat to global health. It is a critical-priority pathogen according to the World Health Organization (WHO) [6].

In Gram-negative bacteria such as *P. aeruginosa*, the cell envelope consists of the cytoplasmic membrane, a periplasmic space, and an outer membrane. The structural complexity of cell requires specialized secretion systems to transport proteins from the cytoplasm to the extracellular environment or directly into host cell [7]. Protein secretion systems are some of the most important virulence factors in *P. aeruginosa*. These secretion systems are molecular machines involved in process such nutrient uptake, antimicrobial resistance (AMR), killing of neighboring bacteria, killing of host cells, that help ensure the survival of its cells during infection [8–10]. *P. aeruginosa* has six different types of secretion systems (Type 1 to Type 6). Every system has distinct structural components, mechanisms of action, and roles in virulence and environmental adaptation [8, 11]. In general, these systems are categorized into one-step mechanisms (Types 1, 3, 4, and 6) and two-step mechanisms (Types 2 and 5). Proteins are translocated directly over entire cell envelope in one-step mechanisms, whereas in two-step mechanisms, proteins first pass the inner membrane and then the outer membrane [10, 12].

For the two steps systems, Sec (general secretion) and Tat (twin-arginine translocation) are fundamental for the initial transport of proteins across the bacterial cytoplasmic membrane [7, 13]. The Sec pathway primarily handles proteins in an unfolded state and guides them through an inner membrane embedded integrated conducting channel, called SecYEG translocon. In contrast, the Tat pathway specializes in translocating fully folded proteins [14]. Proteins that are translocated to the periplasm via either the Sec or Tat pathway can then serve as substrates for other specialized secretion systems such as the Types 2 and 5 systems [7].

In *P. aeruginosa* quorum sensing (QS) orchestrates a wide array of physiological processes, including key components and substrates of the secretion systems [15–17]. This cell-density dependent communication mechanism is composed of four interconnected systems-Las, Rhl, Pqs, and Iqs-which together modulate gene expression in response to population thresholds and environmental cues [18, 19].

The aim of this review is to present a comprehensive overview of the six different secretion systems of *P. aeruginosa*, emphasizing their structure and functional domains, substrates, regulatory networks, and roles in microbial keratitis pathogenicity, as well as the latest advancements in novel therapeutics for *P. aeruginosa* infections by targeting its secretion systems.

### The type 1 secretion system (T1SS) and effectors in *P. aeruginosa*

The Type 1 Secretion System (T1SS) is one of the simplest secretion systems in *P. aeruginosa* and utilizes a one-step secretion mechanism. The T1SS relies on a continuous channel protein to transfer the effector proteins from the cytoplasm to the extracellular space.

**Fig. 1:**
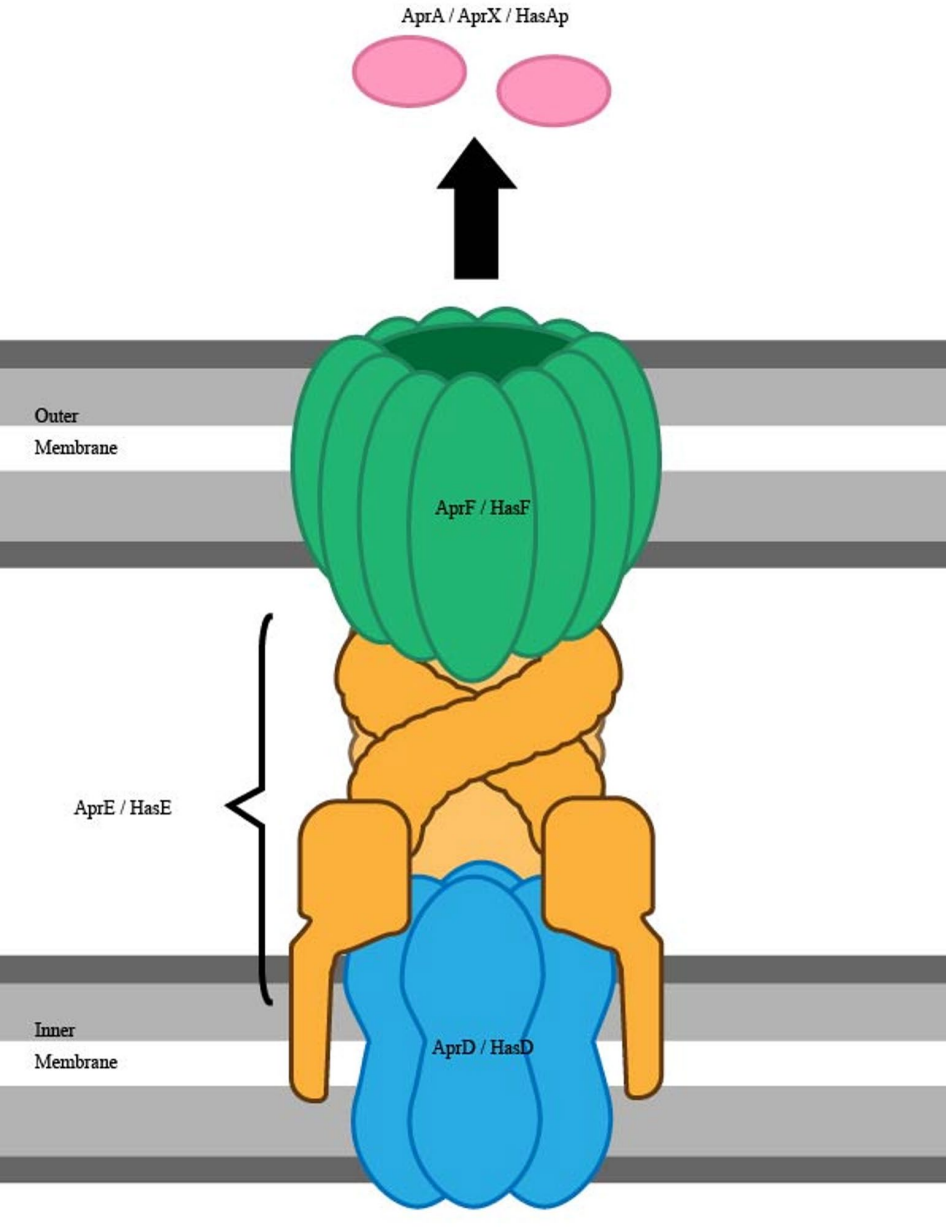
Schematic representation of type 1 secretion system (T1SS) of *P. aeruginosa. *The T1SS has two gene clusters, AprDEF and HasDEF, both of which are structurally similar. They are composed of an ATP binding cassette (ABC) protein - AprD or HasD, the membrane fusion protein - AprE or HasE and the outer membrane protein - AprF or HasF respectively. Being a one-step secretion system, it releases effectors such AprA, AprX or HasAp

The T1SS found in *P. aeruginosa* consists of three main sections, an ATP Binding Cassette (ABC) protein, an inner membrane protein, and an outer membrane protein. There are two known T1SS gene clusters in *P. aeruginosa*, the AprDEF cluster and the HasDEF cluster (Fig. 1). The AprDEF cluster is thought to be specific for two substrates, the alkaline protease AprA and the alkaline metalloprotease AprX. In contrast, the HasDEF system is involved in iron uptake [11]. In the AprDEF cluster, the AprD protein is the ABC protein, with AprE serving as the membrane fusion protein, and AprF being the outer membrane protein. In the case of the HasDEF cluster, HasD serves as the ABC protein whilst HasE and HasF proteins serve as the membrane fusion and outer membrane proteins, respectively. The HasDEF cluster secretes the heme-binding protein HasAp, which captures heme from host hemoglobin [20].

### Regulation of the T1SSs and their effectors

The main transcriptional regulators of *aprA* are LasR, PvrA, σ^SigX^, and σ^AlgT^, with σ^SigX^ also regulating the *aprD*, and *aprX* genes [21]. There are direct LasR binding sites in the *aprA* promoter [21]. The RelA and SpoT mediators of the bacterial stringent stress response regulate the intracellular levels of ppGpp (guanosine tetraphosphate), and ppGpp was found to affect *aprA* gene regulation. In ppGpp-deficient strains *aprA* showed a 9.6-fold downregulation [21]. The *has* gene cluster is regulated by Fur, AmpR, σ^HasI^, σ^HxuI^ [22].

### The role of the T1SSs and their effectors in the pathogenesis of keratitis infection

AprA, whilst not essential for causing eye infections [23, 24] does have a supporting role. After release from cells, AprA causes significant necrosis in corneal tissues [25, 26]. It can break down fibrin, human complement components C1q and C3 [27] and cytokines such as tumor necrosis factor-α and interferon-γ [28]. AprA increases bacterial attachment to the corneal epithelium by exposing receptors [29].

### Therapeutic target of the T1SSs and their effectors

Of the proteins present in the T1SSs, inhibiting AprA, due to its involvement in virulence and ability to combat host defense mechanisms, may have the potential for therapeutic intervention. The two enzymes which mediate the overall response via ppGpp-modulation: RelA and SpoT, may potentially prove to be therapeutic targets as the entire bacterial stringent stress response hinges on their proper functionality [21]. Whilst inhibiting iron uptake mediated by the HasDEF system could reduce bacterial virulence, the many mechanisms *P. aeruginosa* possesses to scavenge iron [30] may limit this as a therapeutic target.

### The type 2 secretion system (T2SS) and effectors in *P. aeruginosa*

The Type 2 Secretion System (T2SS) is a complex nanomachine comprising 12 to 15 distinct protein components, each encoded by a separate gene, that together span the entire cell envelope [31–33]. The T2SS enables many gram-negative bacteria to transport toxins and hydrolytic enzymes such as proteases and lipases from the periplasm across the outer membrane and into the external environment [33]. This occurs after the proteins are first translocated across the inner membrane via the Sec or Tat transport systems [34].

*P. aeruginosa* utilizes two active T2SS, the Xcp and Hxc systems. The Xcp system plays a key role in exporting several virulence factors, including exotoxin A, lipases, phospholipase C, elastase (LasB), and alkaline phosphatase. In contrast, the Hxc system, which was acquired more recently through horizontal gene transfer (HGT) from betaproteobacteria, is specifically involved in the secretion of alkaline phosphatases [34, 35].

The T2SS of *P. aeruginosa* consists of an inner membrane complex, pseudopilus and an outer membrane complex. There is significant structural and functional concordance between the T2SS and type 4 pili, a surface appendage that aids in bacterial motility [32, 33]. The type IV pilus protein, PilA, possesses similarities to the pseudopilins.

**Fig. 2:**
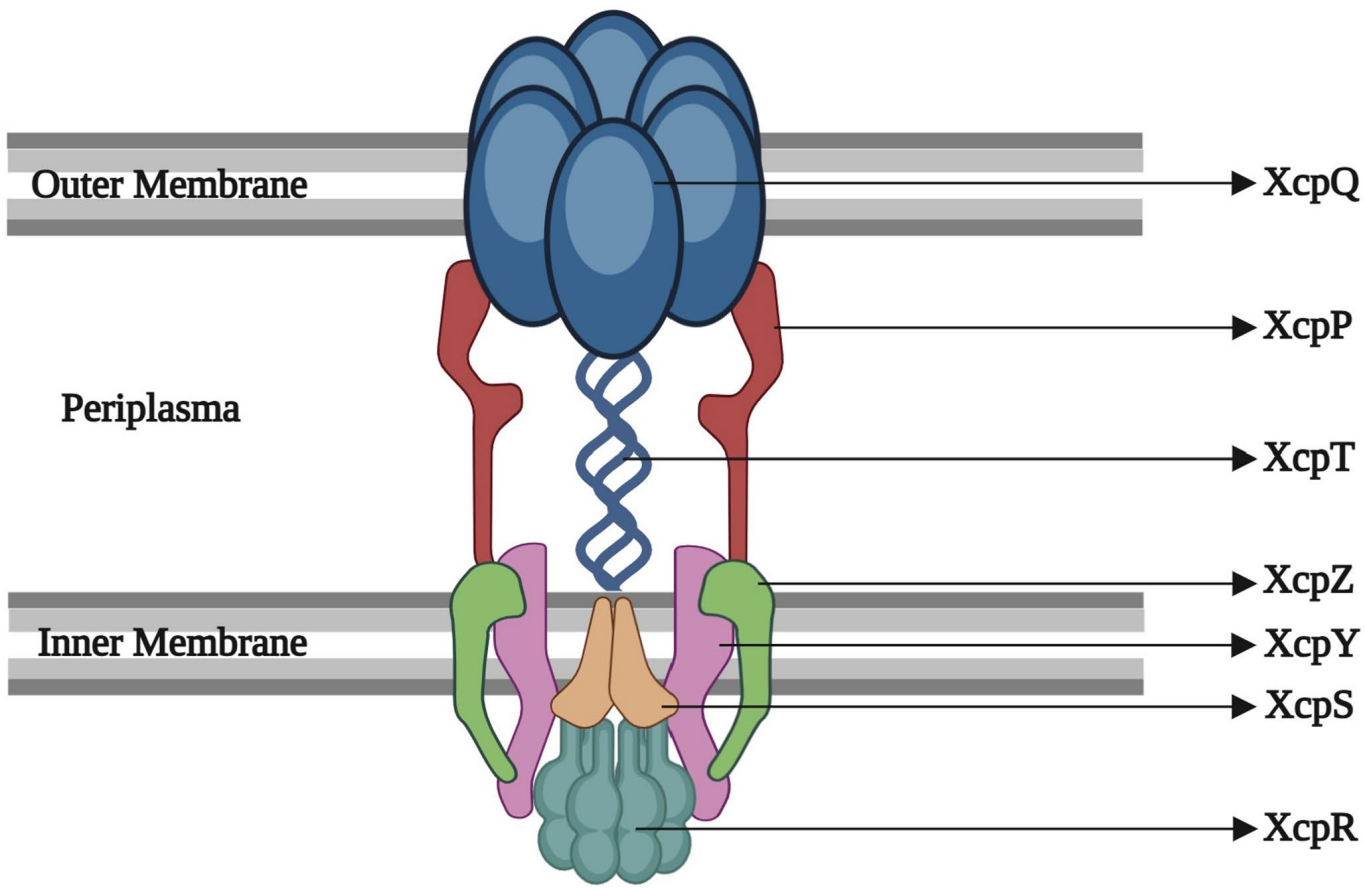
Schematic representation of type 2 secretion system (T2SS) of *P. aeruginosa.* The T2SS consists of an outer membrane complex, pseudopilus and an inner membrane complex. The outer membrane complex is formed by the secretin XcpQ, while the periplasmic pseudopilus components XcpP and XcpT connect the outer and inner membrane subassemblies. The inner membrane consisting of XcpZ, XcpY, and XcpS, provides structural stability and interacts with the cytoplasmic secretion ATPase XcpR, responsible for powering the system

The inner membrane (IM) complex comprises several key proteins XcpP, XcpS, XcpY and XcpZ, including XcpR, a cytoplasmic ATPase essential for the function of the T2SS [31] (Fig. 2). The membrane-associated proteins XcpY and XcpZ serve as anchoring and stabilizing components, coming together with XcpR to form a complex at the cytoplasmic membrane. XcpR is a member of the secretion-associated nucleoside triphosphatase (NTPase) family, characterized by multiple conserved domains spanning from N1 to C2, as well as an additional N0 domain. This IM complex is critical for communication between the pseudopilus, cytoplasmic ATPase, and outer membrane, coordinating their interactions to drive efficient substrate export [7].

The T2 pseudopilus is a multi-fibrillar structure composed of a bundle of two to nine individual pilus fibrils, each approximately 7 nm in diameter [34]. Each fibril is composed of five pilin (XcpT) monomers per helical turn, with the N-terminal regions-oriented inward toward the core of the pilus and the C-terminal regions facing outward toward the external environment [34]. To span the periplasm (~ 20 nm), the pseudopilus likely requires five repeats of an asymmetric unit, each ~ 4.3 nm long [34]. Each unit may contain two XcpT subunits, totaling approximately ten XcpT monomers to reach the periplasmic length [34]. Its relatively short length allows dynamic assembly and disassembly without high energy expenditure. The pseudopilus passes through the outer membrane via XcpQ, a 9.5 nm-wide secretin pore [34]. Pseudopilus elongation may be regulated by minor pseudopilins (e.g., XcpX) that act as capping or termination signals [34]. Under biofilm-forming conditions, type 2 secretion is downregulated, but pseudopilus formation is enhanced. Elevated XcpT expression in biofilms promotes surface adhesion through the formation of elongated and adhesive pseudopili [34].

The outer-membrane complex of the T2SS functions as a channel in the outer membrane that allows the passage of folded periplasmic substrates [7]. This channel is formed by a multimeric protein known as the secretin XcpQ, which has a long N-terminal region thought to extend into the periplasm and interact with T2SS components in the inner membrane [7].

In *P. aeruginosa*, the T2SS secretes several factors contributing to its pathogenicity including exotoxin A, LasA and LasB proteases, as well as phospholipases [32]. Strains lacking both T2SS and Type 3 Secretion System (T3SS) are significantly less virulent, highlighting the importance of these systems in pathogenesis [36].

Exotoxin A (ETA) is a ribosylating enzyme, released into the extracellular milieu by the T2SS, which inhibits host cell protein synthesis [37]. LasA (Staphylolysin) is a 20 kDa zinc-dependent metalloprotease encoded by the *lasA* gene. It is secreted via the T2SS, where it undergoes cleavage from its inactive proenzyme form (pro-LasA) into the mature, active LasA enzyme [38]. As its name suggests, staphylolysin facilitates rapid lysis of *Staphylococcus aureus* and enhances the activity of other proteases, particularly LasB [5]. LasB is a 33 kDa zinc-metalloprotease encoded by the *lasB* gene [38] and synthesized as a 52 kDa precursor protein where it is retained in the periplasm to prevent premature proteolytic activity. After activation and secretion, LasB targets multiple host proteins, including collagen, elastin, fibronectin, laminin, and immunoglobulins, and interferes with early phagocytic clearance of *P. aeruginosa* at wound sites [39, 40]. Protease IV, a serine protease with a molecular mass of approximately 26 kDa, is also secreted via T2SS and acts specifically at the C-terminus of lysine-containing peptides, enabling it to degrade complement proteins, immunoglobulins (IgG), as well as fibrinogen and plasminogen [28, 41].

Phospholipase C enzymes are secreted via the T2SS following export through the Tat pathway [42]. Two homologs exist, the hemolytic form (PlcH) and the non-hemolytic form (PlcN), with expression modulated by phosphate availability [43]. They share approximately 40% sequence similarity at the N-terminal region, while diverging substantially downstream. Both isoforms hydrolyze phosphatidylcholine (PC); however, PlcH also targets sphingomyelin, and PlcN preferentially hydrolyzes phosphatidylserine. PlcH activity predominantly affects eukaryotic membranes and lung surfactants, where PC is abundant. PlcH can also suppress host immune responses by degrading neutrophil membranes [44]. A third phospholipase, PlcB, is secreted via the Sec pathway and hydrolyzes phosphatidylethanolamine (PE) [42].

### Regulation of the T2SS and its effectors

The Las and Rhl systems are hierarchically structured and critical for activating T2SS-secreted exoproducts. The Las system synthesizes N-3-oxododecanoyl-L-homoserine lactone (3O-C12-HSL) via the LasI synthase [15]. This molecule binds to the transcriptional regulator LasR, initiating a QS cascade that controls expression of T2SS substrates such as exotoxin A (ExoA), elastase (LasB), and protease. The Rhl system, driven by N-butyryl-L-homoserine lactone (C4-HSL) and its regulator RhlR, further amplifies the expression of genes encoding T2SS-secreted virulence factors such as LasB elastase and RhlA/B enzymes involved in rhamnolipid synthesis [15]. Protease IV concentrations produced by *lasRI* mutants can be restored to levels comparable to the parent strain following complementation of the quorum-sensing gene deficiencies [45]. The Pseudomonas quinolone signal (PQS) system also integrates into this regulatory network, fine-tuning the expression of LasB and other T2SS substrates through the transcriptional regulator PqsR. Lastly, the integrating quorum-sensing system (IQS) serves as a stress-responsive pathway, compensating for Las system inactivation under hostile conditions such as phosphate limitation or oxidative stress. IQS plays a role in sustaining virulence gene expression, including T2SS components, when other QS systems are compromised [17, 46]. Together, these interdependent QS systems coordinate the regulation of the T2SS, ensuring that virulence factors are secreted in a tightly regulated, population-dependent manner to maximize pathogenic potential while minimizing detection by the host immune system. Hxc, the second T2SS in *P. aeruginosa* which secretes LapA, an alkaline phosphatase, is regulated by phosphate availability in the environment [47].

### The role of the T2SS and its effectors in the pathogenesis of keratitis infection

Protease IV-producing strains caused corneal ulceration in animal models, while non-protease strains (lacking Protease IV) did not [48]. The release of proteolytic enzymes and degradation of secretory immunoglobulin A through the T2SS plays a role in the pathogenesis of keratitis infection through the degradation of ground substance, dispersion of collagen fibrils and stromal weakening which can result in ulceration [48, 49]. In addition, the T2SS had been shown to impair corneal epithelial wound healing [50].

Protease IV is strongly associated with descemetocele formation and can destroy protective protease inhibitors (e.g., α1-antitrypsin, C1-inhibitor), weakening corneal defense. Moreover, Protease IV cleaves lactoferrin, a component of tears involved in the innate immune response [18]. Mutants lacking Protease IV have reduced virulence in mouse keratitis [48]. In the cornea, LasB contributes to disruption of tight junctions and facilitates microbial invasion [51]. It also promotes biofilm formation by regulating rhamnolipid production [52]. Clinically, LasB activity is associated with hypopyon formation and increased corneal ulcer size, directly contributing to disease severity [53].

However, LasA and LasB are not considered key virulence determinants in keratitis [23], as experimental deletion of both *lasA* and *lasB* genes in murine corneal infection models did not result in attenuated disease [23]. Overall, alkaline protease, LasB, and LasA contribute to tissue damage but are not essential for initiating or maintaining corneal infection [23].

### Therapeutic target of the T2SS and its effectors

Understanding the role of T2SS and its secreted products is essential for developing targeted therapies against *P. aeruginosa* infections.​ A number of secreted toxins and invasins are promising targets for the development of antibody therapy which are listed in Table 1.

**Table 1:** Therapeutic targets for the T2SS and its effectors in *P. aeruginosa* [54]

Target	Therapeutic Agent	Mechanism of Action	Development Status
Exotoxin A	Antibodies (incl. toxoid vaccine)	Binds and neutralizes exotoxin A, preventing inhibition of protein synthesis and immune dysfunction	Clinical studies, vaccine trials
Protease IV	Antibody inhibitors	Neutralizes Protease IV, preventing degradation of host immune proteins (Ig, complement, fibrinogen)	Preclinical
Phospholipase C	Antibodies	Neutralizes PLC to reduce membrane damage, immune evasion, and chronic infection persistence	Unknown
LasA and LasB	Antibodies; vaccine candidates	Block cleavage of immune proteins and cytokines; reduce epithelial damage and immune evasion	Preclinical

It may also be feasible to block certain parts of the *P. aeruginosa’s* protein transport system, in particular the T2SS ATPase XpcR. Building on fragment-based inhibitors originally developed against EPEC EscN (a T3SS ATPase), researchers identified a compound that, while designed to inhibit the T3SS, also blocks secretion of Exotoxin A (ExoA) via the T2SS [55]. The compound showed low toxicity in human cells and may serve as a scaffold for future antimicrobial development, offering a promising indirect method to disrupt T2SS-dependent virulence in *P. aeruginosa* [55].

### The type 3 secretion system (T3SS) and effectors in *P. aeruginosa*

The Type 3 Secretion System (T3SS) functions by injecting effector proteins directly into the cytosol of the eukaryotic host cell, thereby promoting disease pathogenesis through enhanced colonization, replication, and survival in hostile environments [56]. A T3SS was identified in *P. aeruginosa* in 1996 [10], but its structure was not visualized until 2005 [57]. The T3SS shares an evolutionary origin with the bacterial flagellar system, although the precise nature of this relationship remains uncertain. Phylogenomic and comparative analyses provided strong evidence that the T3SS evolved through the exaptation of ancestral flagellar components [56, 58].

**Fig. 3:**
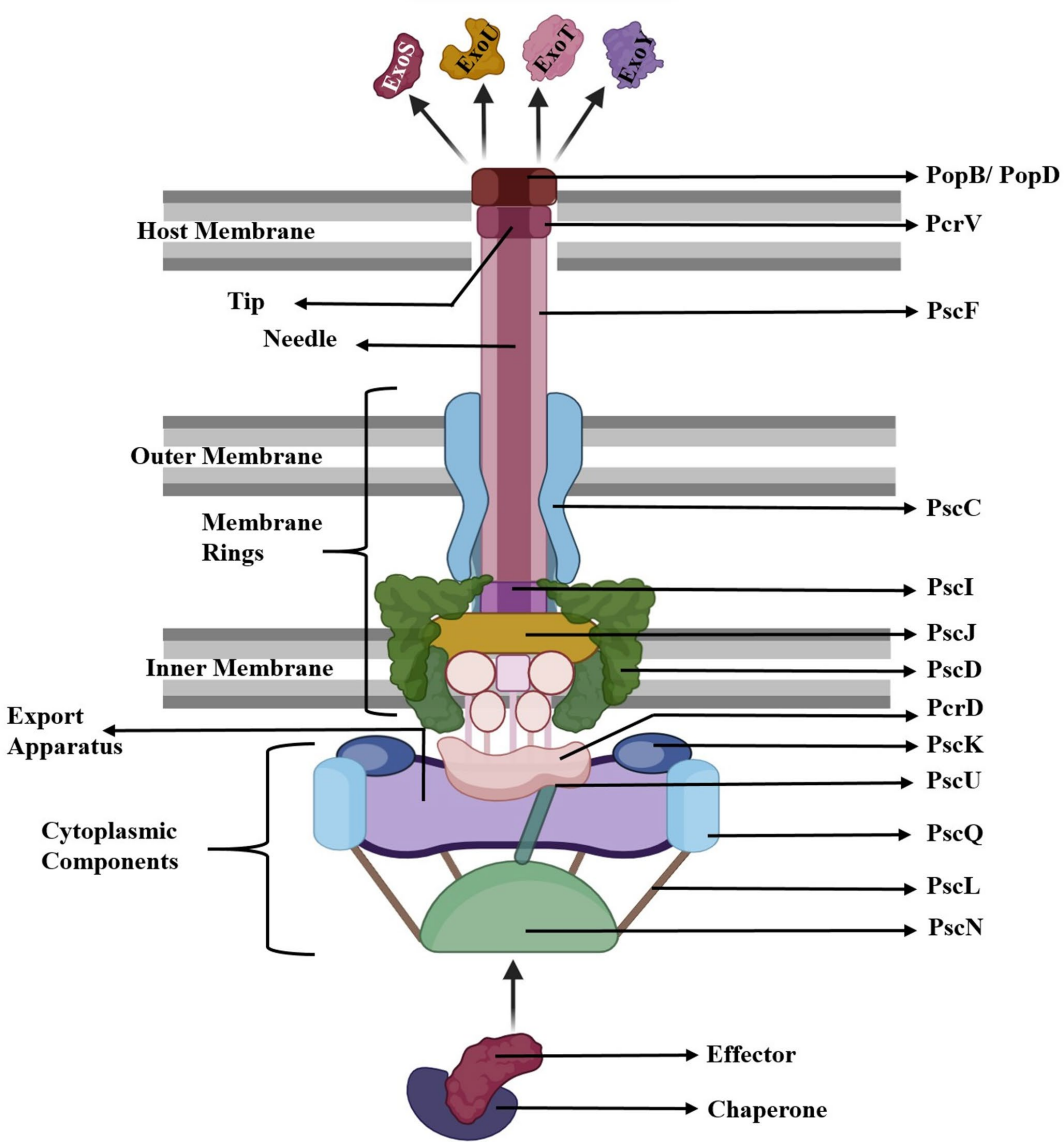
Schematic representation of type 3 secretion system (T3SS) of *P. aeruginosa.* The T3SS is a syringe-like nanomachine composed of a translocation apparatus and a needle complex which includes an extracellular appendix, membrane components (basal body and export apparatus) and cytoplasmic components. The translocation apparatus composed of PopB/PopD interacts with the host membrane to facilitate effector delivery. PcrV assists in the assembly of PopB and PopD proteins or serves to bridge PopB/PopD pores with the needle complex. The needle complex is made of PscF which connects the translocon to the secretion machinery anchored in the bacterial membranes. The basal body comprises the outer membrane ring (PscC) and inner membrane rings (PscJ, PscD) coated by PscI which provides structural support. The export apparatus (PcrD, PscU, PscR, PscS, PscT) coordinates protein passage, while the cytoplasmic components (PscK, PscL, PscQ, and the ATPase PscN) drive substrate recognition, unfolding, and energy-dependent secretion. Effectors such as ExoS, ExoT, ExoU, and ExoY are delivered with the aid of specific chaperones

The T3SS is composed of a complex needle structure, a translocation apparatus, and a regulatory system (Fig. 3). These components are encoded by 36 genes arranged into five consecutive operons (*pscNOPQRSTU*, *popNpcr1234DR*, *pcrGVHpopBD*, *exsCEBA*, and *exsDpscBCDEFGHIJKL*) all located within a specific region of the *P. aeruginosa* PAO1 genome, known as the 55-minute region on the genetic map [56]. Additional genes required for T3SS function, including those encoding effector proteins (*exoS*, *exoT*, *exoU*, and *exoY*) and chaperone proteins (*spcS* and *spcU*), are located elsewhere in the genome or, in some cases, on a pathogenicity island [59]. These effector proteins are not essential for the assembly or secretion activity of the T3SS itself, but they are critical for T3SS-mediated virulence.

The needle complex, which spans from the bacterial cytoplasm to the host cell, can be broadly divided into three main sections namely the extracellular appendix, membrane and cytoplasmic components [56]. The extracellular needle-like structure, measuring 60 to 120 nm in length, is composed of PscF protein subunits arranged in a helical formation. This configuration forms a hollow tube that facilitates the passage of secreted factors [60]. The needle also plays an important role in sensing host cells [57]. The membrane components may be further subdivided into the basal body and export apparatus [56].

The basal body, which serves to transport effector proteins from the bacterial cytosol to the extracellular needle [57], consists of a series of protein rings that span from the inner to the outer membrane of the bacterium [56]. Two distinct outer rings, OR1 and OR2 [61], are composed of the PscC protein, a member of the secretin family that oligomerizes with the lipoprotein PscW to form an outer membrane channel [60]. By contrast, the inner rings are formed from the lipoprotein PscJ and in some cases, PscD as well [56, 57, 60, 61]. PscI forms a cylindrical inner rod within the central lumen of the basal body rings, creating an internal channel that links the multi-ring base to the extracellular needle-like filament and may provide structural support for its assembly [56, 61]. The export apparatus, located in the inner membrane, interacts with the cytoplasmic ring and is composed of five proteins (PscU, PcrD, PscR, PscS, PscT) which play a critical role in the assembly of the T3SS basal body [56, 61].

The cytoplasmic region includes the cytoplasmic ring and ATPase complex [56]. The cytoplasmic ring, otherwise known as the C ring, is formed by the PscQ protein and may regulate or assist with protein secretion. The ATPase complex comprises ATPase (PscN), positive regulator PscQ, negative regulator PscL and cofactor PscK. The role of ATPase (PscN/SctN) in *P. aeruginosa* T3SS is multifold. PscN (SctN) is crucial for T3SS function, and its deletion was found to impair secretion and prevent host cell intoxication [62]. T3SS ATPase has catalytic and mechanical functions. It hydrolyses ATP to recognize and bind exported proteins at the export gate, dissociate chaperone-substrate complexes and unfold secreted proteins to allow passage through the narrow needle channel [62]. ATP hydrolysis mainly facilitates substrate preparation (e.g., unfolding and chaperone removal) and works in conjunction with the proton motive force (PMF), which is the primary driver of substrate translocation through the needle. In addition to this function, the ATPase complex is likely involved in the hierarchical sorting of substrates, beginning with needle subunits, followed by translocators and finally effectors. Interaction with the gatekeeper complex (PopN-Pcr1-Pcr2-PcsB) modulates substrate recognition, suggesting a regulatory role in secretion timing [62].

The translocation apparatus, also known as the needle tip complex, receives effector proteins from the needle complex and injects them into the host cell [60]. The translocation apparatus of *P. aeruginosa* consists of three key proteins, known as translocators: PopB, PopD, and PcrV. The hydrophobic proteins PopB and PopD interact with the host cell membrane to form a transmembrane pore measuring approximately 1–6 nm in diameter [5, 56, 60]. The hydrophilic protein PcrV is essential for translocation but is not part of the pore itself. PcrV is believed to either create a multimeric scaffold that assists in the assembly of PopB and PopD proteins or otherwise serves to “bridge” the needle complex to already formed PopB/PopD pores, resulting in a continuous conduit through which effectors may travel [60]. Interestingly, the translocation pore can induce host cell death even in the absence of effector proteins, through both direct and indirect mechanisms [56]. Directly, the pore disrupts membrane integrity, increasing membrane permeability and causing osmotic changes that may lead to osmotic shock [56]. Indirectly, it activates host defence mechanisms that contribute to cell death [63]. The translocation apparatus triggers the release of pro-inflammatory cytokines through the activation of the nod-like receptor (NLR) family, CARD domain containing 4 (NLRC4) inflammasome [63]. Activation of this inflammasome complex results in the conversion of pro-inflammatory cytokines, IL-1β and IL-18 into their active forms ultimately leading to cell death through pyroptosis [63].

Four secreted effector proteins have been identified, namely ExoU, ExoS, ExoT, and ExoY which is notably fewer than in many other T3SSs [2]. The expression of these proteins varies by strain and is influenced by the site of infection [64]. ExoY and ExoT are found in approximately 85–90% and 92–100% of isolates, respectively [65, 66]. By contrast, ExoU and ExoS which are associated with cytotoxic and invasive phenotypes respectively, are typically expressed in a mutually exclusive manner with ExoS present in about 58–72% of strains and ExoU in 28–42% [66, 67].

ExoU is a large, 74 kDa protein encoded on a genomic island and acquired through HGT [68]. It is highly cytotoxic and has been associated with more severe disease phenotypes and worse outcomes in both human and animal models [68]. The ExoU toxin has phospholipase A2 activity, and upon translocation into the host cell, disrupts the host cell’s plasma membrane [69]. It remains distinct from other effector proteins due to its ability to cause rapid lysis of epithelial cells. The ExoU protein consists of several distinct domains including an N-terminal chaperone-binding domain (residues 55–101), a patatin-like phospholipase domain (residues 106–471), a C-terminal bridging domain (residues 480–580), and a membrane localization domain (residues 588–687) [68]. ExoU requires a number of eukaryotic cofactors for activation including ubiquitin, phosphatidylinositol 4,5-bisphosphate [PI(4,5)P₂] and superoxide dismutase 1 (SOD1) [68].

ExoS is a 453-amino-acid bifunctional protein [65]. It contains a GTPase-activating (GAP) domain that inactivates small GTPases such as Rac and Cdc42, leading to transient disruption of the actin cytoskeleton and cell rounding [57]. These GTPases typically cycle between an active GTP-bound state and an inactive GDP-bound state [57]. The ExoS protein was found to catalyze the hydrolysis of GTP, locking the GTPases in their inactive GDP-bound form and thereby disrupting actin organization. ExoS utilizes an arginine finger (Arg146) to stabilize transition states and maintain GTPase activity [57]. It also includes an ADP-ribosyltransferase (ADPRT) domain that modifies host proteins like Ras [57]. While the ADPRT activity was historically thought to be dependent on activation by Factor Activating ExoS (FAS), a 14-3−3 protein whose binding site spans residues 418–429, more recent evidence suggests that it acts more as a chaperone or scaffolding factor that facilitates ExoS activity [57]. The ADPRT domain leads to broad host cell dysfunction including cell death, actin disassembly, inhibition of DNA synthesis, endocytosis, and vesicular trafficking as well as disruption of cell-to-cell junctions [57]. ExoS interferes with innate immune responses with the ADPRT domain working to block phagocytosis and NADPH oxidase activity in neutrophils [70]. It also induces incomplete NETosis through CitH3 formation and nuclear breakdown [70]. ExoS suppresses NLRC4 inflammasome activation while paradoxically promoting IL-1β secretion via NLRP3 [70].

The ExoT protein is widespread presence of in nearly all strains of *P. aeruginosa* [71]. The ExoT protein shares 76% amino acid sequence identity with ExoS, indicating a high degree of homology [60]. Both are characterized as bifunctional proteins possessing N-terminal GAP activity (residues 78–235) and C-terminal ADPRT activity (235–457), requiring host cell cofactor 14-3−3 for activation [60]. ExoT intoxication is sufficient to induce apoptosis, although it occurs more slowly, often beyond 5 h post-infection, compared to the cytotoxic effects of ExoS, which typically manifest within 2 to 5 h post-infection [71]. Both active domains are implicated in ExoT cytotoxicity by targeting differing apoptotic pathways. Unlike ExoS, ExoT ADP-ribosylates a small number of targets, primarily Crk-I and Crk-II (CT10-regulator of kinase adaptor proteins) [5]. This specificity is likely conferred by a unique α-helix region that is notably absent in ExoS strains. The inhibition of the Crk signaling pathway leads to a number of effects including disruption of Rac1 signaling, reorganization of the actin cytoskeleton, blockage of cytokinesis and inhibition of cell migration, adhesion and proliferation [5]. This has serious consequences for host defence, ultimately delaying wound healing and increasing *P. aeruginosa’s* ability to cause opportunistic infections by disrupting epithelial barriers and inhibiting phagocytosis [5].

ExoY is a 42 kDa nucleotidyl cyclase that, upon activation by F-actin, produces cyclic nucleotide monophosphates (cNMPs) such as cyclic GMP (cGMP), cyclic UMP (cUMP) and to a lesser extent, cyclic AMP (cAMP) and cyclic CMP (cCMP) within host cells [72, 73]. ExoY promotes membrane bleb-niche formation in epithelial cells through its adenylate cyclase activity; however, this is not associated with increased bacterial intracellular replication [74].

Exotoxins secreted via the T3SS are associated with specific chaperones. ExoS and ExoT share the chaperone SpcS, whereas ExoU is paired with its unique chaperone, SpcU. ExoY lacks an associated chaperone protein. These chaperones assist in directing effector proteins to the ATPase complex and play a crucial role in unfolding them, a step essential for their effective secretion [60]. They also play a role in maintenance and storage.

### Regulation of the T3SS and its effectors

The regulation of the T3SS in *P. aeruginosa* is a complex process involving four main regulatory proteins including ExsA, ExsC, ExsD, and ExsE [75]. The regulation operates at two interconnected levels, namely the initiation of secretion and the subsequent transcription of T3SS genes. Transcription is induced only when secretion is triggered, typically after the bacterium makes contact with a host cell [75].

The T3SS genes are regulated at the transcriptional level by ExsA, which belongs to the AraC family of transcriptional activators. ExsA binds to an adenine-rich consensus element located upstream of the − 35 RNA polymerase binding site (often called TNAAAANA) [59]. This facilitates gene transcription and effector expression under low-calcium conditions [76, 77]. Under non-secreting conditions, such as in the presence of high extracellular calcium, T3SS is repressed at both the transcriptional and secretion levels [76, 77]. ExsA is inhibited by the anti-activator ExsD, which binds to the ExsA N-terminal effectively blocking ExsA-dependent transcription. ExsC, dubbed an “anti-anti-activator,” sequesters ExsD interfering with the formation of the ExsA1-ExsD1 complex, thereby allowing ExsA to activate the transcription of the genes necessary for the T3SS [59, 78]. Upon activation of secretion by the bacterium contacting with host cells, ExsE which controls the ExsC is exported through the T3SS needle complex [77]. As intracellular ExsE levels fall, ExsC is freed to bind ExsD, thereby releasing ExsA to initiate transcription of T3SS genes [59, 77].

Beyond the ExsA regulatory cascade, T3SS gene expression is modulated by important global regulatory systems that respond to environmental stimuli including the cAMP-Vfr pathway and GacS/GacA two-component system [59, 79].

The adenylate cyclases, CyaB and to a lesser extent CyaA, increase intracellular cAMP levels in response to environmental signals [59, 79]. Elevated cAMP levels activate Vfr, a global regulator homologous to the *E. coli* cAMP receptor protein (CRP), thereby affecting the expression of various genes tied to T3SS and other virulence factors [60]. Studies have revealed that mutants deficient in cAMP or Vfr show diminished expression of numerous genes, indicating that the T3SS is integrated into a larger regulatory network for pathogenicity [59, 79].

The GacS/GacA two-component system involves two sensor proteins, RetS and LadS. This system influences the expression of small regulatory RNAs (sRNAs) that can modulate T3SS gene expression [59]. The sensor kinase RetS promotes acute infection traits by inducing T3SS genes and repressing genes associated with biofilm formation. Conversely, LadS acts reciprocally by downregulating T3SS expression and promoting chronic infection traits, such as biofilm development [80].

### The role of the T3SS and its effectors in the pathogenesis of keratitis infection

The exotoxins ExoU and ExoS, delivered via the T3SS, have been recognized as key contributors to the pathogenesis of keratitis [81, 82]. *P. aeruginosa* strains expressing the *exoS* gene (invasive strains) invade epithelial cells through the breakdown of tight junctions [83], while strains with the *exoU* (cytotoxic strains) gene kill host cells [84]. Consistent with these functional observations, transcriptomic analyses of *P. aeruginosa* isolates from keratitis patients have revealed a significant upregulation of T3SS effector genes *exoU* (log₂FC = 18.57) and *exoS* (log₂FC = 2.27), compared to isolates from healthy conjunctival sacs, indicating that T3SS is actively expressed during corneal infection [85].

In the cornea, the T3SS of bacteria possessing the *exoU* gene is activated upon contact with the host cell or in the presence of low extracellular calcium [75]. This process is controlled by the ExsA regulatory system and functions to inject effector proteins like ExoU into host cells [75]. ExoU is secreted in an inactive form bound to chaperone protein, SpcU at the N terminal. SpcU stabilises ExoU, guides it to T3SS, and prevents bacterial self-toxicity. Upon injection into the host cell cytosol, ExoU binds to eukaryotic co-factor ubiquitin and becomes activated. It then localises to the plasma membrane via phosphatidylinositol 4,5-bisphosphate (PIP_2_), subsequently undergoes oligomerisation and degrades the plasma membrane exerting phospholipase (PLA_2_) activity [75]. Since PIP_2_ co-activates ExoU and boosts its phospholipase activity in vitro when ubiquitin is present, it is believed that ExoU uses PIP_2_ to anchor to the plasma membrane and more effectively hydrolyse nearby phospholipid substrates. This culminates in rapid loss of membrane integrity and necrosis the epithelial cell. Early changes include actin cytoskeleton collapse and focal adhesion detachment. In addition to cell lysis, ExoU modulates host signaling pathways. ExoU-positive strains were found to activate c-Jun NH2 terminal kinase (JNK) mitogen-activated protein kinase (MAPK) pathway resulting in increased synthesis of IL-8, an important pro-inflammatory chemokine that promotes neutrophil recruitment [75]. This may induce a hyper-inflammatory response, which aids tissue damage and immune subversion [75].

Upon contact with the corneal cell, ExoS-positive *P. aeruginosa* uses its T3SS to invade corneal epithelial cells, interacts with scaffold protein IQGAP1, affecting actin and zonular occludin 1 (ZO1) to reduce tight junction integrity thereby facilitating bacterial invasion and tissue penetration [86]. They replicate within plasma membrane blebs [86] which act as replication niches and were found to enhance intracellular persistence and bacterial proliferation protecting from immune surveillance. All of which are thought to eventually lead to corneal cell death with features of apoptosis or necrosis [86].

In the context of keratitis, the *exoU* positive (cytotoxic) genotype was more frequently observed in contact lens wearers, with a prevalence of 54% in a sample of 28 contact-lens wearers, compared to just 11% in a group of 27 non-contact lens wearers [82]. While in non-contact lens-related microbial keratitis, the prevalence of *exoS* strains (70–80%) is more common than *exoU* strains [87]. Thus, in contact lens-related corneal infections, cytotoxic strains predominate over invasive strains and tend to cause more severe infections [81].

Keratitis in contact lens wearers has been linked to biofilm formation on both the contact lens and its storage case [82]. The ExoU-positive genotype correlates with strong biofilm production and more resistant to commonly prescribed antibiotics, whereas the ExoS-positive genotype is linked to weak to moderate biofilm formation and lower resistant to antibiotics [82, 88, 89]. Additionally, cytotoxic strains were found to be more resistant to contact lens disinfectants [90], particularly RenuFresh even when administered for the recommended time [91], perhaps suggesting that the higher prevalence of cytotoxic strains among contact-lens wearers can be attributed to the selectivity of commercially available solutions [86].

In the SCUT trial, cytotoxic strains were associated with poorer initial visual acuity compared to invasive strains, likely due to greater corneal oedema (Borkar et al., 2013). Interestingly, these patients showed greater visual improvement at the 3-month follow-up. Additionally, the therapeutic response to corticosteroids appeared diminished in infections caused by cytotoxic strains, likely because their suppression of the inflammatory cascade reduces the availability of steroid-responsive targets.

### Therapeutic target of the T3SS and its effectors

The growing understanding of the structure and function of the T3SS has led to the identification of several promising therapeutic targets and strategies. Drugs have been found that target various aspects of the T3SS, including its expression, ATPase activity, needle and translocon apparatus, basal body, effector proteins, and host immune response [67]. With highly conserved structure among gram-negative bacteria [56] drugs that target the T3SS may be highly advantageous as numerous pathogens will be susceptible to secretion system inhibitors. Additionally, as secretion systems are a virulence mechanism not critical for bacterial survival, the selective pressure to develop resistance is significantly reduced [92, 93]. Some novel therapies specifically targeting the T3SS of *P. aeruginosa* including their mechanism of action and development status are summarized in Table 2:

**Table 2:** Therapeutic targets for the T3SS and its effectors in *P. aeruginosa* [67, 94]

Therapeutic Agent	Mechanism of Action	Development Status
Plant Phenolic Compounds	Modulate the GacS-GacA two-component system, reducing *exoS* transcription	Preclinical
N-Hydroxybenzimidazoles	Inhibit the DNA-binding activity of ExsA, the master regulator of T3SS gene expression	Preclinical
Salicylidene Acylhydrazides	Interfere with T3SS transcription, possibly by targeting regulatory pathways	Preclinical
Hydroxyquinolines	Inhibit the secretion of *exoS* and targets flagellar motility	Preclinical
Thiazolidinones	Target the basal body of the T3SS, disrupting its assembly and function. Hypothesized to target secretin which is a protein implicated in the T2SS, T3SS and Type 4 pili	Preclinical
Phenoxyacetamides	Bind to the needle protein PscF, inhibiting T3SS needle assembly.	Preclinical
Monoclonal Antibodies (e.g., KB001)	Target PcrV, a component of the T3SS translocation apparatus, preventing effector delivery into host cells	Clinical trials (Phase II)
Pseudolipasin A (PSA), (2-[3-chlorophenyl) amino]−4,6-dimethylnicotinamide, 2-[(2,5-dichlorophenyl) amino]−4,6-dimethylnicotinamide	Inhibit ExoU-mediated cytotoxicity in HeLa cells and HCE-T scratch assay; reduce cell lysis and wound size in a dose-dependent manner	Preclinical

### The type 4 secretion system (T4SS) and effectors in *P. aeruginosa*

Type 4 secretion system (T4SS) is a complex, multi-subunit nanomachine found in both Gram-positive and Gram-negative bacteria. This system shares evolutionary ancestry with bacterial conjugation machinery [95]. The T4SS apparatus forms a complex that spans both the inner and outer bacterial membranes. Unlike some other secretion systems, T4SS is capable of translocating both proteins and nucleic acid across bacterial or eukaryotic cell membranes [96].

T4SS is classified into two main subtypes: Type 4 A (T4ASS) and Type 4B (T4BSS) based on their architecture and function [10, 97–99]. In Gram negative bacteria, T4ASS is implicated in DNA transfer, contributing to HGT and the dissemination of antibiotic resistance genes [95, 99, 100]. On the other hand, the T4BSS is similar to the Dot/Icm system in *Legionella pneumophila*, which is primarily associated with the translocation of effector proteins into eukaryotic host cells [101]. Thereby, these systems are critical for modulating host immune responses and promoting bacterial survival within host environments [100, 102]. In addition to translocation, T4SS form a pili like surface structure [103].

In addition to the well-characterized Type 4 A and Type 4B secretion systems, *P. aeruginosa* possesses a diverse group of T4SSs classified as “other T4SSs”. This third group is encoded by genomic islands (GIs) such as *clc*, pKLC102, PAPI-1/2 (*P. aeruginosa* pathogenicity island), and the PAGI (*P. aeruginosa* genomic island) [98, 104]. These GI-T4SSs form a phylogenetically distinct lineage and are associated with integrative and conjugative elements (ICEs). These ICEs are capable of self-encoded excision, circularization, replication, and conjugal transfer [10, 98, 99, 105]. The “other T4SSs” group contributes to genome plasticity and the acquisition of antibiotic resistance and virulence enabling bacterial survival in various environments, including clinical settings [98, 104, 106]. For example, the *clc*, pKLC102, and PAPI families encode T4SSs that facilitate the dissemination of antibiotic resistance and virulence determinants [98, 107–110]. Genomic analyses have identified that the PAGI family (e.g., PAGI‑2, PAGI‑4, PAGI‑5) further diversify the T4SS in *P. aeruginosa* and help in interbacterial interactions and pathogenesis [104, 109, 111, 112]. The ongoing research and advancements in genomic sequencing are likely to reveal even more diverse T4SS types in the future.

**Fig. 4:**
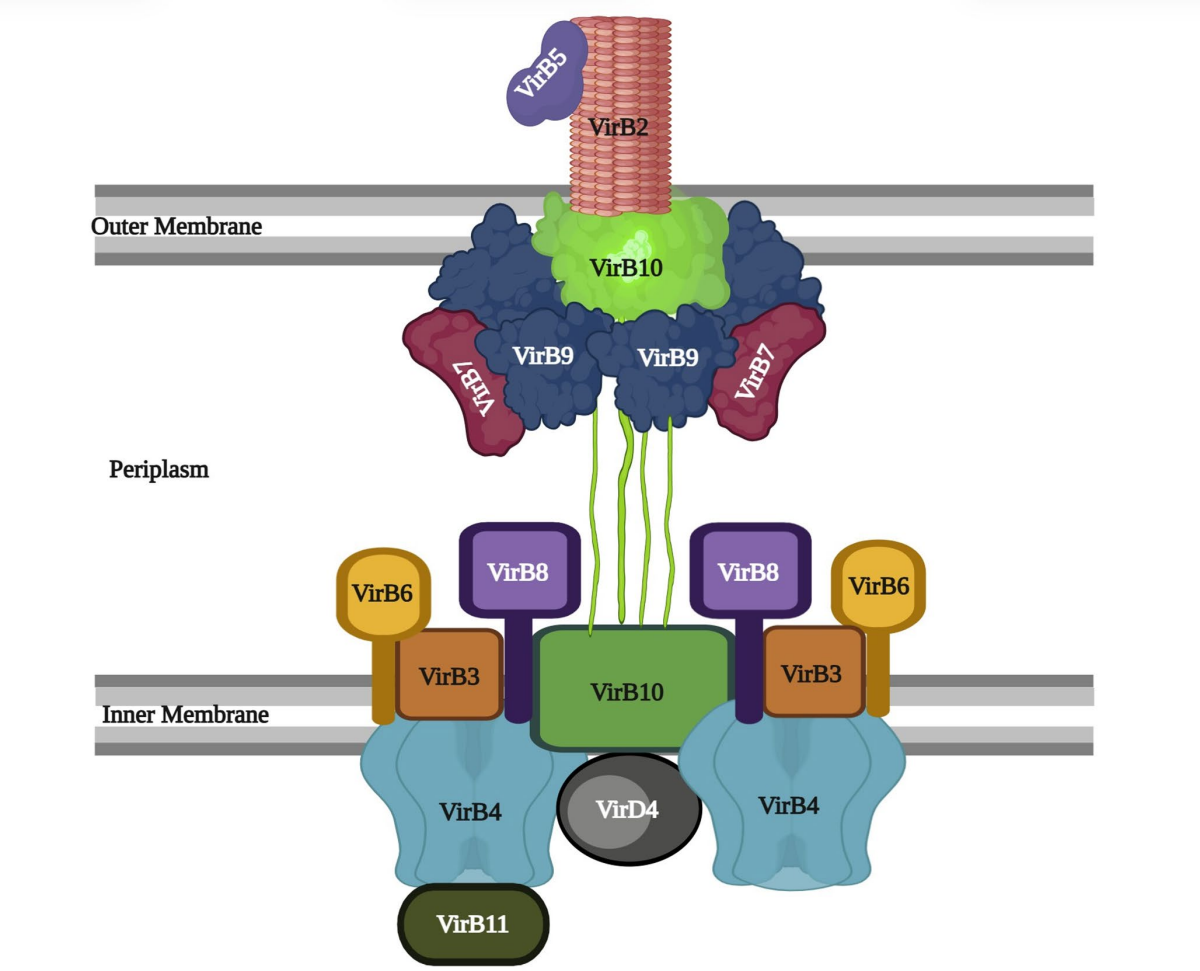
Schematic representation of type 4 secretion system (T4SS) of *P. aeruginosa.* The T4SS structure includes an outer membrane core complex, an inner membrane complex and a pilus-like extension. The extracellular pilus is formed by VirB2 and stabilized by VirB5, enabling contact with target cells. The outer membrane core complex, composed of VirB7, VirB9, and VirB10, spans the periplasm and connects to the inner membrane subassemblies. The inner membrane platform, including VirB3, VirB6, VirB8, and VirB10, provides structural support and coordinates substrate passage. Energy for substrate transfer is supplied by the ATPases VirB4, VirB11, and VirD4, which facilitate substrate recruitment and translocation

Some T4SSs are termed as minimized as they consist of only the core components, while others may require up to 25 different proteins for assembly and function [103, 113]. Structurally, a prototypical T4SS consists of three major subassemblies: the outer membrane core complex, the inner membrane complex and the pilus like extension (Fig. 4) [103, 113].

The outer membrane core complex (OMCC) forms a channel by connecting the inner and outer membranes. This barrel-shaped structure is primarily composed of homologs of VirB7, VirB9, and VirB10 proteins. They carry substrate through the periplasm and across the outer membrane (OM) [100]. The VirB7, a lipoprotein, targets the core complex to the OM, whereas VirB9 regulates substrate selection and T-pilus biogenesis. The VirB10 is present in both membranes and acts as a sensor protein to transduce energy signals to initiate substrate transfer [114].

The inner membrane complex (IMC) transfers substrates across the inner membrane of the bacterial cell. This complex includes ATPase proteins (VirD4, VirB4 and VirB11) and scaffolding proteins (VirB3, VirB6, VirB8). VirD4 is a highly conserved ATPase that functions as a receptor to mediate recruitment of substrates. Meanwhile, the two additional ATPases (VirB4 and VirB11) provide the energy for substrate transport through the system [99]. The scaffolding proteins (VirB3, VirB6, VirB8) are attached with the N-terminal domains of VirB10 in the IMC. These proteins are crucial for the assembly and function of the secretion system. Furthermore, VirD4, VirB4, VirB11, VirB3, and VirB8 localize independently at the cell poles, while VirB6 protein localize dependently within the IMC [99, 114].

The pilus-like structure emerges from the bacterial cell surface, and it is made up of two main protein subunits: the major pilin (VirB2) and the minor pilin (VirB5). This extracellular structure helps bacterial attachment, biofilm formation, and virulence by transferring genetic material into bacterial cells [99, 100, 114, 115]. The VirB2 forms the structural core of the pilus by repeating thousands of major pilin subunits, assembling into a helical fiber. The subunits form a conserved pilin fold with an extended N-terminal α-helix and a C-terminal globular β-sheet domain. The minor pilin (VirB5) is located at the pilus tip. Although minor pilin protein is less abundant than VirB2, they play a vital role in pilus elongation and function as an adhesin for host-cell recognition [114, 116].

The T4SSs in *P. aeruginosa* are not well- known for secreting effector during infection [68, 117]. However, recent proteomic studies suggest that T4SSs can manipulate cellular processes, enabling the secretion of effector proteins in both eukaryotic and prokaryotic cells [118].

### Regulation of the T4SSs and their effectors

The T4SSs expression are tightly regulated by environmental cues and regulatory proteins. The GacS/GacA two-component system helps this bacterium to response to environmental stress by adjusting the activity of T4SSs. This system connects signals such as nutrient level and oxidative stress to regulation of virulence factors secretion, allowing them to adapt more effectively during infection. *P. aeruginosa* also influences its secretion systems expression in response to environmental factors like nutrient availability, oxygen levels, and host-derived signals. For an example, low iron level down-regulates the expression of T4SSs genes, whereas high iron availability promotes their expression [119]. The post-translational regulation provides an additional layer of control. The T4SSs machinery are subject to phosphorylation-dependent activation which mediates by kinases such as PpkA [120].

### Role of the T4SSs in the pathogenesis of keratitis infection

The role of T4SSs in keratitis pathogenesis appears minimal or uninvestigated. The T4SSs are primarily involved in HGT through pilus like extension, enabling the exchange of plasmids and genomic islands between bacterial cells. This process promotes bacterial adaptation by contributing to genome plasticity and helps dissemination of antibiotic resistance genes and novel virulence factors across bacterial populations. The acquisition of resistance and virulence genes through HGT can make keratitis infections much harder to treat with standard antibiotics [103, 118].

### Therapeutic target of the T4SSs and their effectors

Targeting T4SSs has significant therapeutic potential because of their function in bacterial pathogenicity and AMR dissemination. Thus, addressing T4SSs is an urgent and developing novel therapeutics should be a top priority. Small-molecule inhibitors that interfere with the assembly of the T4SSs machinery can block its translocation activity. Targeting essential ATPases (VirB4, VirB11) or core structural components could be an effective strategy [103, 118]. A previous study reported that 2-imino-5-arylidentiazolidinone has a wide spectrum of action against gram-negative pathogens. This molecule can decrease the mobility of T4SSs pili, suggesting they may prevent T4SSs from functioning in *P. aeruginosa* [121]. Another potential therapeutic target can be interfering with the regulatory pathways, such as quorum sensing. This mechanism can indirectly reduce the virulence by inhabiting the synthesis of T4SSs components. A QS inhibitor called furanones has the potential to down-regulates T4SSs expression, which would lessen bacterial pathogenicity without causing resistance [122].

### The type 5 secretion system (T5SS) and effectors in *P. aeruginosa*

The type 5 secretion system (T5SS) of *P. aeruginosa* is a Sec-dependent, two-step secretion pathway functioning as a virulence factor via contributions to numerous roles including biofilm formation, adhesion as well as proteolysis [123]. It consists of several subtypes including the autotransporters (T5aSS), the two-partner passenger-translocators (T5bSS), the trimeric autotransporters (T5cSS), the hybrid autotransporters (T5dSS) and the inverted autotransporters (T5eSS) [47]. It operates by first transporting proteins across the inner membrane via the Sec export machinery and then translocating them past the outer membrane either via a C-terminal β-barrel translocator domain or another helper protein [124]. The system is responsible for the transport of many different protein types referred to as autotransporters (ATs) [10]. The key effectors of this system include EstA, an esterase found to contribute to virulence by altering extracellular rhamnolipids, influencing biofilm formation, and modulating twitching; swimming and swarming motility [125] and PlpD, a phospholipase with a patatin-like domain and β-barrel.

**Fig. 5:**
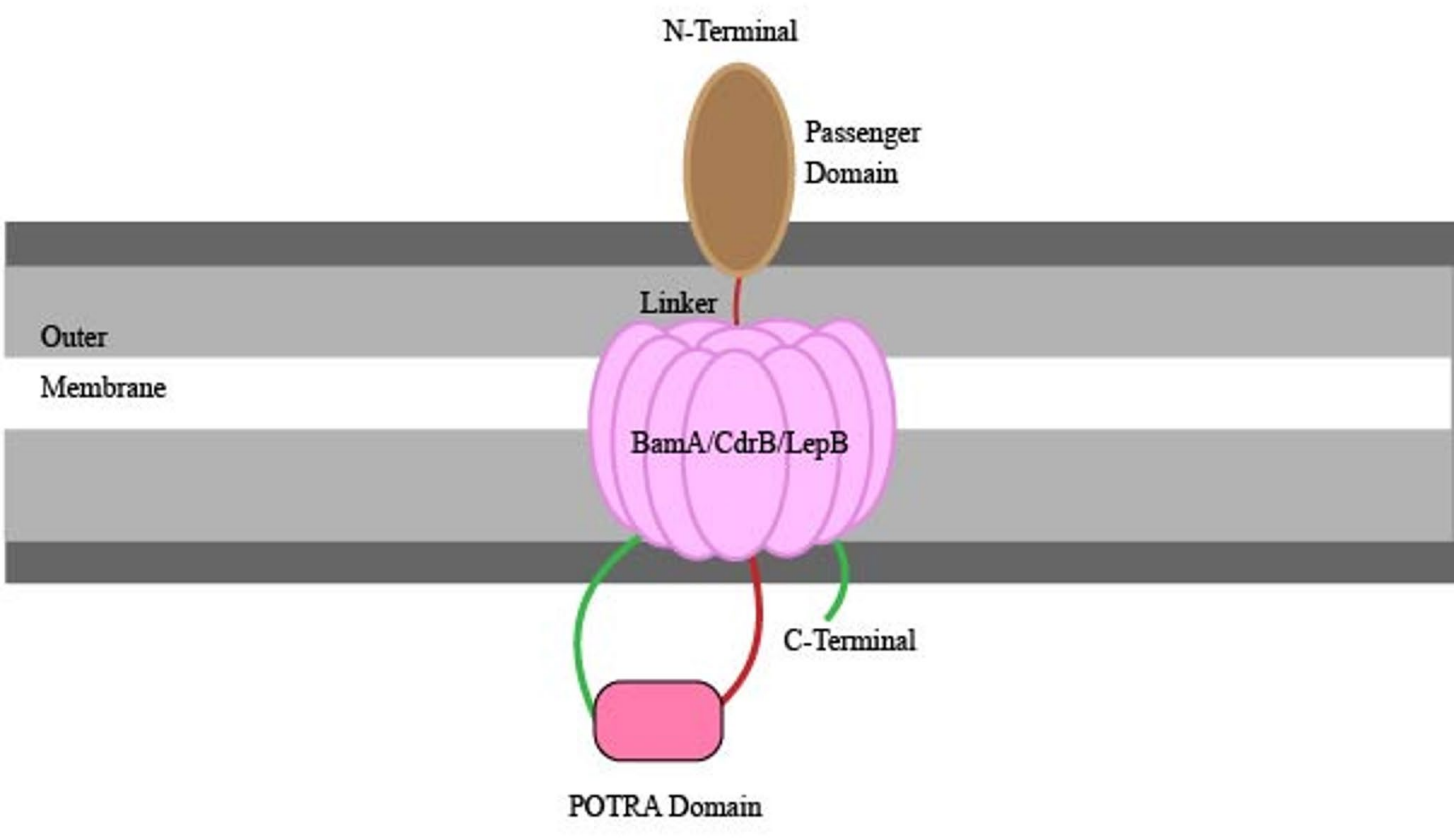
Schematic representation of type 5 secretion system (T5SS) of *P. aeruginosa.* The T5dSS is an autotransporter known as patatin-like protein D, PlpD (lipolytic enzyme), which consists of an N-terminal signal, a passenger domain which is a homodimer with phospholipase A1 activity and a C-terminal based β-barrel translocator domain (BamA or CdrB or LepB). A single periplasmic polypeptide transport associated domain (POTRA) connects the N-terminal PlpD passenger domain and the C-terminal based β-barrel translocator domain

The T5SS has a tripartite structure consisting of an N-terminal signal, a passenger and β-barrel translocator domain [123] (Fig. 5). Once past the inner membrane, signal peptidases in the periplasm cleave the signal sequences allowing chaperone proteins such as Skp, SurA, and DegP to usher the proteins to the BAM complex (β-barrel assembly mechanism) within the outer membrane. The C-terminal β-barrel translocator domain is inserted into the outer membrane under the regulation of the BAM complex and the TAM complex (translocation and assembly module), creating a pore that allows the proteins to exit past the outer membrane to the cell surface [10, 123]. In *P. aeruginosa*, one of the key autotransporters is the T5dSS, commonly known as patatin-like protein D, PlpD, which acts as a lipolytic enzyme. Its passenger is a homodimer with phospholipase A1 (PLA1) activity. It has been characterized as a hybrid autotransporter due to the fused nature of the two distinct polypeptides encoded in one operon, i.e. the single periplasmic polypeptide transport-associated domain (POTRA) and the β-barrel which is composed of 16 β-strands. PORTA connects the C-terminal β-barrel to the N-terminal PlpD passenger with additional support from a short linker bound to the passenger’s domain. Post-translocation, the passenger is cleaved for release into the extracellular space [10, 126]. A recent study challenges the delivery of the passengers via the translocation process as energetically unfavorable and identifies that the PL-domain resides exclusively in the periplasm with PlpD forming a homodimer [127]. Another prominent classical autotransporter (Type 5a) is the EstA, a lipase of *P. aeruginosa* which cleaves rhamnolipids, contributing to biofilm maturation [128]. Its passenger domain adopts an α/β-hydrolase fold, making it structurally unique in comparison to other β-helical structures [129]. Other major proteins involved in T5SS virulence include CupB5, TpsA, TpsB and the proteases.

### Regulation of the T5SSs and their effectors

The activity of T5SSs virulence factors is tightly regulated by various transcriptional and post-translational mechanisms. EstA is regulated by ExsC and ExsD, components that are typically associated with T3SS regulatory networks but also influence T5SSs effector production. The adhesin CupB5 is controlled by AlgU and MucA, central regulators of alginate biosynthesis and biofilm maturation, along with TpsB4/LepB, which mediate its translocation and assembly overproduction [22, 123]. The TpsA and TpsB regulations involve outer membrane assembly factors and secretion pathway components to ensure proper localization and function.

### Role of the T5SSs and their effectors in the pathogenesis of keratitis infection

Generally, the esterase, EstA enhances rhamnolipid production, cell motility as well as biofilm formation [128]. Another protein is the chaperone-usher pathway pili - CupB5, a major adhesin which activates alginate overproduction [10, 22, 129]. TpsA and TpsB are two β-barrel outer membrane exoproteins which enhance bacterial adhesion whilst aiding in immune escape by secreting large virulence proteins [44]. The role of these secreted proteins (EstA, CupB5, TpsA and TpsB) in the pathogenesis of keratitis has not yet been examined, warranting future investigations.

### Therapeutic target of the T5SSs and their effectors

Therapeutic development against the T5SSs would require targeting the autotransporter’s cleaved domains, with EstA, LepA, and PlpD being the most potential targets [54]. Additionally, as T5SSs supports biofilm formation, there may be potential in identifying anti-biofilm forming molecules.

### The type 6 ecretion system (T6SS) and effectors in *P. aeruginosa*

The Type 6 Secretion System (T6SS) of *P. aeruginosa* is a protein nanomachines utilizing contractile spikes to inject effectors into targets via cell-to-cell contact. The genomes of the *P. aeruginosa* strains encode four different T6SS loci named H1-T6SS, H2-T6SS, H3-T6SS, and H4-T6SS. The newly discovered H4-T6SS is uncharacterized as of yet, however phylogenetic analysis shows it is most related to H2-T6SS [117].There are around twenty toxic effectors involved with the T6SS that are responsible for metabolic impairment, cell wall disruption and nucleic acid degradation [130].

**Fig. 6:**
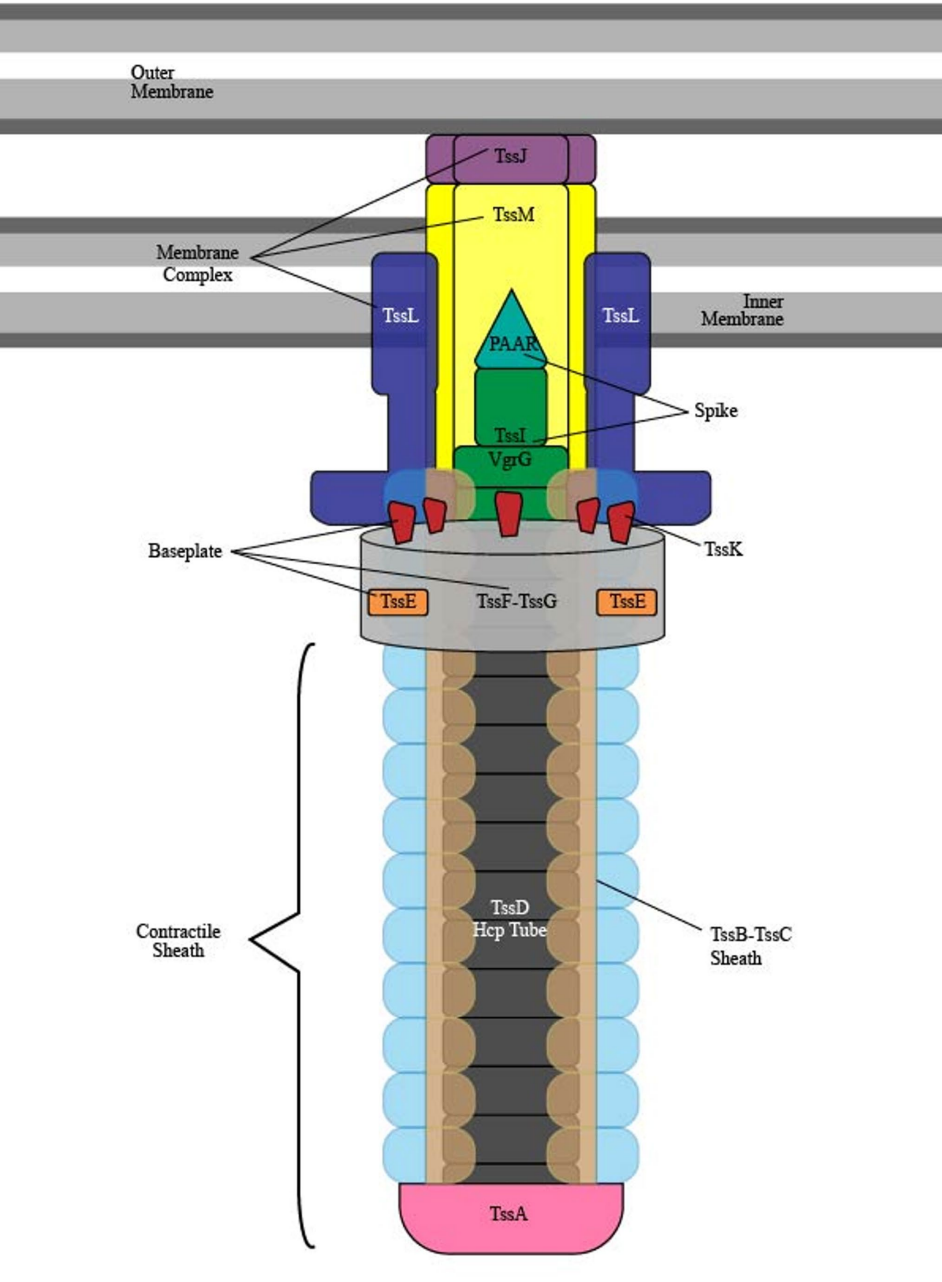
Schematic representation of type 6 secretion system (T6SS) of *P. aeruginosa.* The T6SS consists of a membrane complex, a baseplate, and a contractile sheath with a spike. The membrane complex is composed of the structural proteins TssJ, TssM and TssL. The baseplate is made up of the structural proteins TssK, TssE, TssF, TssG and it is connected to the membrane complex and contractile sheath. The sheath is composed of TssB-TssC proteins surrounding the inner spike. The spike’s tip is made of the PAAR (Proline-Alanine-Alanine-Arginine) repeat protein and the VgrG (Valine glycine-repeat protein G) trimer/TssI with the inner spike tube composed of Hemolysin coregulated protein - Hcp (TssD) located within the contractile sheath

The T6SS mainly consists of three main structural parts - the membrane Complex; the baseplate; and the contractile sheath containing the spike (Fig. 6). The membrane complex is composed of the components - TssJ, TssM and TssL [131]. TssJ is a lipoprotein, located nearer to the outer membrane, where it protrudes into the periplasm [132] and interacts with the inner membrane protein TssM. Both the TssM and TssL proteins are involved in encoding homologs of T4SS stabilizing proteins [133]. The complex acts as a conduit for effector protein export [134].

The baseplate is made up of the TssK, TssE, TssF, TssG structural components. It is present in the cytoplasm, docked between the membrane complex and contractile sheath, and predicted to be a nucleation point for spike polymerization [134]. A stable complex is formed from two TssF, one TssG and two more TssK trimers, which go on to oligomerize with the tip of the spike component. Based on the components ration of the stable complex (2:1:2), it is proposed that the entire baseplate structure is that of a 6 (TssE), 12 (TssF), 6 (TssG) with 18 (TssK) subunits ultimately connecting to the Membrane complex [135].

The sheath is composed of the TssC-TssB components surrounding an inner spike, with TssA located at the distal end for stabilization. The contractile sheath is anchored to both the baseplate and membrane complex, and contracts to release the spike. The spike’s tip is made up of VgrG (Valine glycine-repeat protein G) trimer known as TssI and a PAAR (Proline-Alanine-Alanine-Arginine) repeat protein. Within the sheath, the inner spike tube is composed of the secreted protein, Hemolysin coregulated protein - Hcp (TssD) [133], which form hexameric rings in the shape of a hollow tube and help bind effectors [136]. There is also an ATPase known as TssH (ClpV) which is involved in the translocation of Hcp and disassembly of the T6SS for refiring [131]. It has been suggested that the DUF2169 accessory proteins which are structurally similar to PAAR domains, function as molecular chaperones to maintain the VgrG-PIPY spike complex prior to export [134].The effectors involved with T6SS do not contain linear signal sequences for recognition but rather rely upon the physical association with the Hcp, VgrG, or PAAR proteins of the spike in order to facilitate transition [137]. In particular, Hcp has shown to utilize the interior of the hexameric rings to accommodate its interacting effectors for accumulation prior to secretion, almost like a chaperone; whilst the VgrG-linked effectors rely on specific chaperones such as DUF2169, DUF4123 and DUF1795 proteins [137].

The structure of the distally located TssA proteins (TssA1, TssA2 and TssA3) also plays a critical role in the contraction process. As seen when the H1-T6SS contracts rapidly whilst the H2-T6SS sheaths maintain their extended conformation. This variation in the TssA structure may also affect its interaction with accessory components like TagA and TagB as well as in domain organization [138].

Of the T6SS present in *P. aeruginosa*, the effectors used can be categorized as either cargo or specialized effectors, with the H1-T6SS primarily responsible for bacterial targets whilst the H2-T6SS and H3-T6SS operate as both bacterial and eukaryotic cell targeting agents. The effectors are paired with their respective immunity proteins which provide the bacteria with self-protection from the effector’s actions [131, 139].

Of note is the sheer number of known effectors acting across the three T6SS secretion systems. There are several effectors of the H1-T6SS system, ranging between Tse1 through to Tse8 [117, 140]. Of these effectors, Tse1 and Tse3 degrade peptidoglycans, Tse2 helps induce quiescence (dormancy) by inhibiting cell proliferation, Tse4 and Tse5 depolarize cell membranes, Tse6 hydrolyzes NAD(P)^+^, Tse7 acts as a nuclease and Tse8 targets transamidosome to halt protein synthesis. However, it was noted that in certain strain subsets, other effectors were also expressed by the H1-T6SS such as Tas1 in place of Tse6 showing potential for more effectors that have yet to be identified [117, 140–142].

For H2-T6SS, most effectors act as Type 6 lipase effectors (Tle) targeting specific organelles [143]. Tle4 acts on the endoplasmic reticulum of eukaryotes, whilst Tle5a (PldA) is cargo effector responsible for bacterial internalization into non-phagocytic cell [139, 144] and killing of neighboring bacterial cell. Interestingly, Tle1 presents with phospholipase A2 toxicity and Tle3 presents with lipolytic active. There are VgrG gene clusters associated with H2-T6SS, of which the metalloprotease VgrG2b interacts with the microtubule γ-TuRC complex to force entry into non-phagocytic cells [139, 145]. RhsP2 has been experimentally categorized as an ART toxin capable of both anti-prokaryotic and anti-eukaryotic killing via ADP-ribosylation of RNA [143]. And the Azu cargo effectors aids in Cu^2^ + acquisition [144].

Finally, the H3-T6SS has three known effectors, Tle5b (PldB), TepB and TseF. Like PldA, PldB acts on both host cell and other prokaryotic cell while TepB and TseF seemingly do not function as killers. TepB has recently been shown to function in biofilm formation as a protease and is potentially involved in the virulent nature of the *P. aeruginosa* strain PA14 Dd [146]. TseF has shown to be involved in iron uptake, specifically iron bound to the quorum sensor Pseudomonas quinoline signal (PQS). This PQS is noted to integrate with the outer membrane forming OMVs (Outer Membrane Vesicles) which later form complexes with extracellular Fe^3+^ ions, this complex formation allows TseF to both bind and pull the complex towards the receptor FptA and the outer membrane porin OprF to facilitate iron uptake, aided by PQS’s inner membrane transporters [147].

The effectors can be grouped based on either function or targets, for example nucleases breaking down DNA/RNA, cell wall degrading amidases and phospholipases targeting inner cell membrane. Additionally, the effector genes are usually present near the T6SS cluster and the orphan islands of *hcp*, *vgrG* and PAAR genes [131].

### Regulation of the T6SSs and their effectors

The *P. aeruginosa* microbe is bile-tolerant and harnesses the positive regulation of bile on quorum sensing to be more infectious via increased promoter activity of T6SSs, efflux pump expression, and resistance to antibiotics [148]. The AmrZ transcription factor has been shown to positively regulate H1 and H3-T6SSs while RsmA represses all T6SSs clusters. And it was shown that mutating *icmF3* gene resulted in impaired motility, without affecting expression of the flagella regulon [149].

Beyond AmrZ and RsmA acting as a global regulator of the T6SSs apparatus [150, 151], both GacA and RpoN positively regulate T6SSs; GacA through the expression of RsmY and RsmZ small RNAs, and RpoN through HcpA and HcpB expression [152]. Most of the effectors are counteracted by their cognate immunity protein, with the only recent finding coming of NfxB potentially acting as a regulator of Tse3 [153]. Regulators of other effectors toxins are presented in Table 3.

**Table 3:** Known regulators of the T6SSs effectors

Effector	Regulator	Reference
Tse1	RsmA via Gac/Rsm Cascade	[154]
Tse2	RsmA, Tsi2	[154]
Tse3	RsmA, NfxB	[153]
Tse4	Gac/Rsm	[154]
Tse7	PAAR–VgrG1b interface residues, Tsi7	[155]
Tle3	Tli3	[156]
Tle4/TplE	Putative ArsP permease (potentially)	[157]
Tle5a/PldA	Tli5a, blaIMP genes (potentially)	[158]
VgrG2b	RsmA	[151]

### Role of the T6SSs and their effectors in the pathogenesis of keratitis infection

In the host cell, PldA activates the phosphatidylinositol 3-kinase (PI3K)/Akt pathway, driving bacterial uptake into cells that would not normally engulf bacteria promoting chronic infections [139, 144]. A study reported over 50% of eye isolates possessed the *pldA* gene [159], however, to date, no specific reports have addressed the function of *pldA* in keratitis or eye infections. Given its prevalence in ocular isolates, this highlights an important gap in current research and suggests that future studies should investigate whether *pldA* contributes to the pathogenesis, virulence, or chronicity of *P. aeruginosa* keratitis.

### Therapeutic target of the T6SSs and their effectors

A trivalent vaccine, POH, composed of the extracellular V-antigen (PcrV) of T3SS in combination with the outer membrane protein I (OprI) and the T6SS’s central component - Hemolysin co-regulated protein (Hcp1), was shown to induce better protection than single components acting separately on murine models. The vaccine significantly reduced acute infection in mice, with the vaccine inducing POH-specific antibody production [160].

Targeting the TssK-TssG protein interface of the T6SSs baseplates, a group of researchers designed a cyclic biomimetic peptide (BCP) capable of binding in the TssK pocket to inhibit T6SSs activity [161]. They validated their in silico construct using nuclear magnetic resonance (NMR) experiments, which found the BCP to significantly interfere with the TssKFGE formation in *E. coli*, but may see a broader application in other T6SSs housing microbes including *P. aeruginosa*, due to the conserved nature of the TssK-TssG protein interface [161].

## Conclusion

*P. aeruginosa* secretes an arsenal of toxins and its pathogenesis are complex because of its virulence factors. Among the different secretion systems and the effectors toxins, ExoU and ExoS secreted from the T3SS are one of the major virulence factors contributing to severe keratitis infections. However, in order to develop anti-virulence therapeutics against this bacterium it is important to know the role of these toxins and others secretion systems along with their regulations in keratitis infection, which are gaps in the current literature. Further studies are needed to know the prevalence of secreted toxins and their role in keratitis by knockout of the specific genes from the *P. aeruginosa* in keratitis.

## Data Availability

Data is provided within the manuscript.
